# The Appraisal of Adults with Congenital Heart Disease: Lesson from Comparison of Surgical Outcomes

**DOI:** 10.1007/s00246-024-03517-6

**Published:** 2024-05-27

**Authors:** Alessandro Giamberti, Paolo Ferrero, Federica Caldaroni, Alessandro Varrica, Giulia Pasqualin, Fabio D’Aiello, Emma Bergonzoni, Marco Ranucci, Massimo Chessa

**Affiliations:** 1 Department of Congenital Cardiac Surgery, IRCCS Policlinico San Donato, University Hospital, Via Morandi 30, 20097 San Donato M.se, MI Italy; 2 ACHD Unit - Pediatric and Adult Congenital Heart Centre, IRCCS Policlinico San Donato, San Donato M.se, MI Italy; 3 Department of Cardiovascular Anesthesia, IRCCS Policlinico San Donato, San Donato M.se, MI Italy; 4 Cardiac Surgery Unit, Department of Cardiac, Thoracic, Vascular Sciences and Public Health, University of Padua, Padua, Italy; 5 UniSR - Vita Salute San Raffaele University, Milan, Italy

**Keywords:** Adult with congenital heart disease, Cardiac surgery, Grown-up congenital heart, Cardiac surgery reintervention

## Abstract

The population of adults with congenital heart disease (ACHD) is constantly growing. There seems to be a consensus that these patients are difficult to manage especially if compared to patients with acquired heart disease. The aim of this study is to compare outcomes and results of cardiac surgery in ACHD patients with a reference population of adults with acquired cardiac disease. Retrospective study of 5053 consecutive patients older than 18 years hospitalized for cardiac surgery during a 5-years period in our Institution. Two groups of patients were identified. Group I: 419 patients operated for congenital heart disease; Group II: 4634 patients operated for acquired heart disease. In each Group were identified low, medium, and high-risk patients, according to validated scores. Right ventricular outflow tract surgery was the most frequent procedure in Group I, while coronary artery by-pass grafting was the most common in Group II. Patients with ACHD were younger (37.8 vs. 67.7 years), with higher number of previous operations (32.1% vs. 6.9%), had longer post-ICU hospital stay (11 vs. 8 days) but had lower ICU stay (1 vs. 2 days), shorter assisted mechanical ventilation (12 vs. 14 h) and lower surgical mortality (1 vs. 3.7%) (all p < 0.001). No differences were found in term of post-operative complications (12.4 vs. 15%). The surgical treatment of ACHD patients can be done with excellent results and if compared with acquired cardiac disease patients they have better results with shorter ICU stay and lower mortality.

- Key question: Are surgical ACHD patients so complex if compared with acquired heart disease population?
- Key findings: the surgical treatment of ACHD patients has excellent results and, compared with a reference population of patients with acquired cardiac disease, they have a lower complexity score and better overall outcomes.
- Take-home message: the majority of ACHD patients can be surgically treated with excellent results and if compared with acquired cardiac disease have shorter ICU stay and lower surgical mortality.

## Introduction

Early diagnosis, advances in surgical and interventional techniques, and medical therapy have increased the overall survival to adulthood of new-borns affected by moderate to complex congenital heart disease (CHD), therefore we are now facing the issue of a new and growing population [1]. These patients, in fact, may have special needs and consequently elevated costs. Recent data estimate that in Europe the current ACHD population accounts for 2.3 million of patients, which significantly outnumbers the pediatric CHD figure of 1.9 million patients [2, 3]. The adults with congenital heart disease (ACHD) population are growing both in term of age [4–6] and number of surgical procedures performed [7]. However, due to its rapidly and constantly evolving figure and features, we lack a complete assessment of the real burden of this population on outcomes and surgical results. Available data are limited and the conclusions from all studies seem to agree that ACHD patients are high risk patients with high occurrence of post-operative complications, longer ICU and in-hospital stay [8–13]. A better understanding of the actual complexity of these patients is mandatory for planning the management of resources and investment, and for a better risk stratification.

In this study, we analyse hospital performance and results for ACHD patients undergoing cardiac surgery and compare them to a reference population of adults operated on for all types of acquired cardiac disease in the same period.

## Materials and Methods

We conducted a retrospective analysis on 5053 consecutive adult patients (older than 18 years) hospitalized for cardiac surgery during 5-years period (January 2015 to December 2019) in our institution.

We decided to end the data collection in December 2019 as the global COVID-19 pandemic has dramatically altered access to treatment for elective patients in most hospitals.

This study was approved by the local Ethical Committee (54/int/2022) Consent was waived due to the retrospective nature of the study. Collection of data was obtained from records and administrative files.

We identified 2 groups of patients: patients operated on for congenital heart disease (Group I-ACHD) and patients operated on for acquired heart disease (Group II-Acquired).

In Group I, ACHD surgical procedures were defined as those performed for a cardiac defect present from birth, except for Bicuspid Aortic Valve (BAV) disease, who were included in the group up to a maximum age of 35 years, in accordance with the National Congenital Heart Disease Audit Website in United Kingdom [14]. Patients with patent foramen ovale (PFO) were excluded.

For each group of patients, we recorded: number of cases, mean age, type of surgical procedure performed, number of redo cases, preoperative risk score category, hospital stay, Intensive Care Unit (ICU) stay, length of assisted mechanical ventilation, complications, and mortality.

In order to evaluate the distribution of patients in terms of risk categories, we used EUROSCORE II [15] for both Groups and we stratified the non-ACHD patients as follows: < 2 = low risk; 2–6.99: medium risk; ≥ 7: high risk. This classification was not defined in the original EuroSCORE II manuscript, and is somehow arbitrary; however, these classes or close to these classes are often used in risk analyses [16]. In Group I, we added the PEACH Score Points [17] also, categorizing the adult patients with congenital heart disease in 3 classes: low, medium, and high risk.

According to the Society of Thoracic Surgeons risk-adjusted operative mortality published in 2015, complications have been defined as occurrence of any one or more of the following: cardiac reoperation, deep sternal infection, permanent stroke, prolonged ventilation (> than 48 h), and renal failure [18].

### Statistical Analysis

Categorical data are reported as number (%). Continuous data are reported as mean (standard deviation) or median (interquartile range) in case of normal or non-normal distribution, respectively. A Pearson’s chi-square test was applied to compare differences between categorical variables, a Student’s t-test for comparison between continuous, normally distributed variables, and non-parametric tests for comparison between continuous, non-normally distributed variables.

A P value < 0.05 was considered significant. A computerized statistical program (SPSS, Inc, Chicago, IL) was used for all calculations.

## Results

### Group I: Adult Patients with Congenital Heart Disease

Between 2015 and 2019 a total of 419 adult patients with congenital heart disease were operated on in our institution. Mean age was 37.8 years (± 14 years). Details on the surgical procedures are summarized in Table 1. Pathology of the right ventricular outflow tract (RVOT) was the most frequently treated (32%), including 100 surgical pulmonary valve implantations, 25 RV-PA conduit replacements, and 8 Tetralogy of Fallot repairs. Atrial septal defect (ASD) closure, including secundum, primum, and sinus venosus repair was performed in 108 patients (26%).

**Table 1:** Cardiac surgical procedures performed in Group I and Group II

Group I (ACHD)—N = 419		Group II (non-ACHD)—N = 4634	
Procedure	N (%)	Procedure	N (%)
Atrial septal defect closure	108 (26)	Isolated valve procedure	1530 (33)
Pulmonary valve implantation	100 (24)	Isolated coronary revascularization	1298 (28)
Aortic valve surgery	38 (9)	CABG + valve	556 (12)
RV to PA conduit replacement	25 (6)	Multiple valve procedure	371 (8)
Tricuspid valve surgery	25 (6)	Ascending aorta procedure	232 (5)
Mitral valve surgery	20 (4.5)	CABG + LV aneurysm	185 (4)
Sub-aortic obstruction surgery	16 (4)	Cardiac tumors	20 (0.4)
Fontan conversion/revision	13 (3)	Others	442 (10.2)
Partial atrio-ventricular defect repair	13 (3)		
Ascending aorta surgery	10 (2.5)		
Tetralogy of Fallot repair	8 (2)		
Others	43 (9)		

Pathology of the left ventricular outflow tract (LVOT) was present in 15.5% of cases, including 38 interventions for aortic valve disease, 16 sub-aortic obstruction relief, and 10 Bentall procedures. Less frequently, tricuspid valve repair/replacement was performed in 6% of patients, mitral valve repair/replacement in 4.5%, Fontan conversion/revision in 3%.

**Fig. 1:**
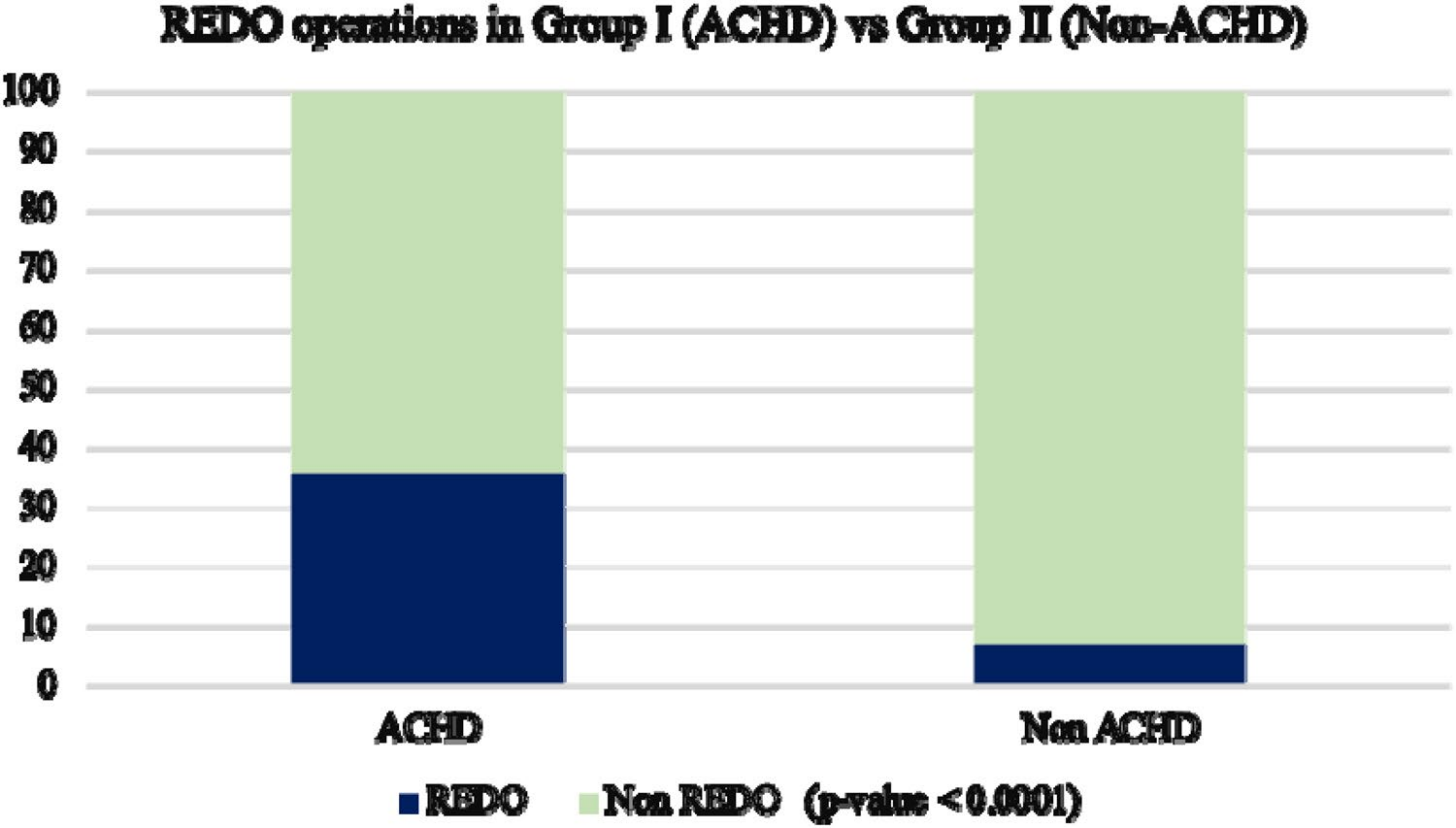
Comparative data on reoperations (%) in both Groups. ACHD: adult congenital heart disease

According to the type of operation performed, 267 patients (63.8%) had primary repair, while 152 patients (36.2%) had a reoperation after previous procedures (Fig. 1). Active endocarditis was present in 16/418 pts in the ACHD group (vs 146/4635 (3.1%) pts in the non-ACHD group, p-value 0.467).

**Fig. 2:**
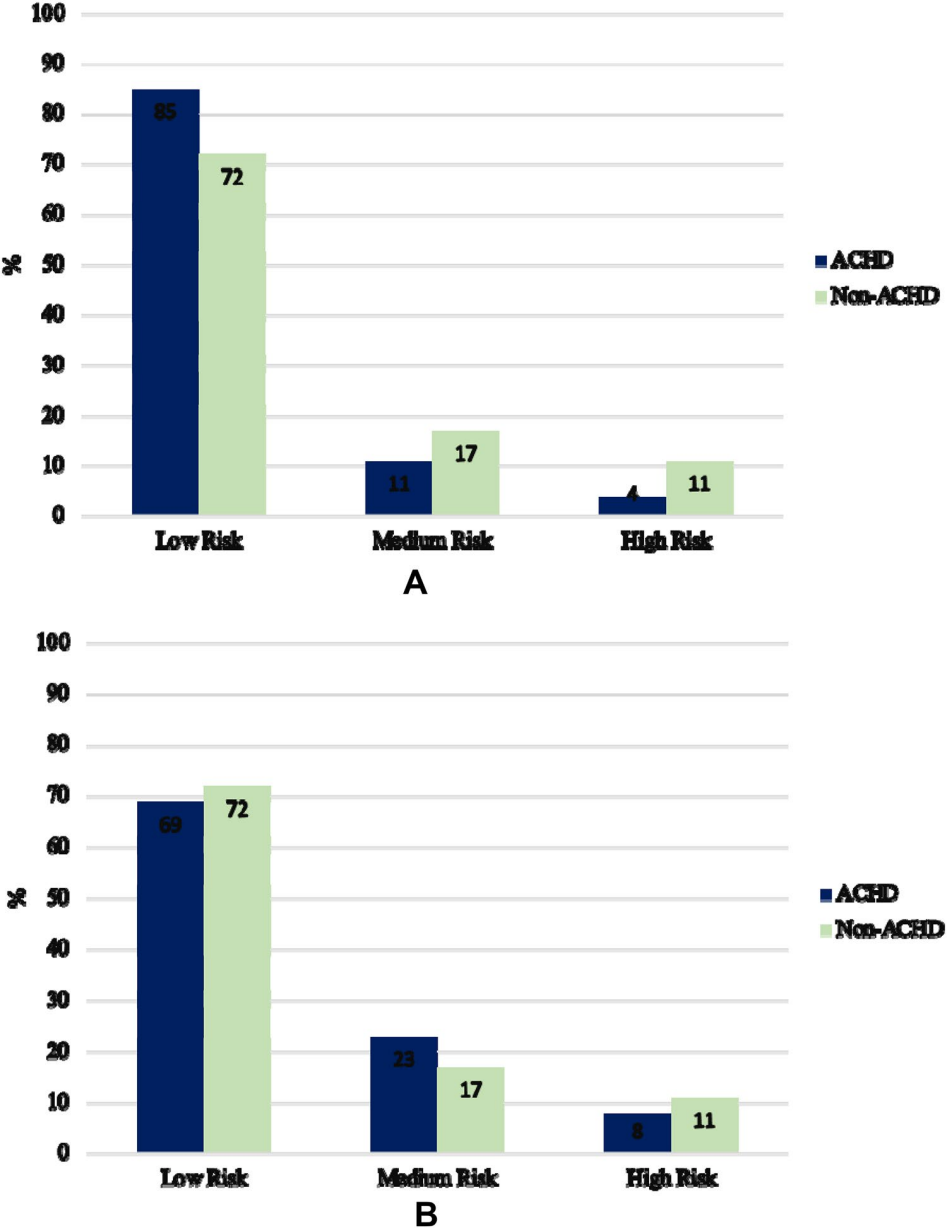
Distribution of the population (%) in terms of risk, according to Euroscore II (**A**) and Euroscore II/PEACH score (**B**). **A** Distribution of the population (ACHD, non-ACHD) in terms of risk, according to Euroscore II. ACHD: adult congenital heart disease. **B** Distribution of the population (ACHD, non-ACHD) in terms of risk, according to Euroscore II/PEACH. ACHD: adult congenital heart disease

To evaluate the distribution of patients in terms of risk, we used the EUROSCORE II [15]. In this group of patients, we also used the most recent and specific PEACH Score Points [16] categorizing the adult patients with congenital heart disease in 3 classes: low risk (point 0), medium risk (points 1), and high risk (points ≥ 2). There were no substantial differences in the two evaluation scores. Most patients had low or medium risk (404 = 96%) and only 15 patients (4%) were included in the high-risk category (Fig. 2, Panel A and B).

In-hospital mortality was 1% (n = 4 patients). Median total postoperative hospital stay was 11 days (8–15) including: a median ICU stay of 1 days (range 1–3), and a median time of assisted mechanical ventilation (AMV) of 12 h (range 7–16) (Table 2).

**Table 2:** Baseline characteristics and outcomes in Group I and Group II

Variable	Group I (ACHD, N = 419)	Group II (Non-ACHD, N = 4634)	p-value
Age (years)	37.8 (14)	61.7 (32)	0.001
Redo Surgery	152 (32)	322 (7)	0.001
Number previous surgeries	1 (1–2)	1 (1–1)	0.001
Low-risk patients	356 (85)	3362 (72)	0.144
Medium-risk patients	48 (11)	812 (18.1)	0.001
High-risk patients	15 (4)	460 (9.9)	0.464
Operative mortality	4 (1)	170 (3.7)	0.001
ICU stay (days)	1 (1–3)	2 (1–4)	0.001
Mechanical ventilation (h)	12 (7–16)	14 (10–18)	0.001
Postop hospital stays (days)	11 (8–15)	8 (7–12)	0.001
Major morbidity	52 (12.4)	730 (15)	0.078

Data are number (%) or mean (standard deviation) or median (interquartile range). ACHD: adult congenital heart disease; ICU: intensive care unit; h: hours; postop: postoperative

Major morbidity occurred in 52 patients (12.4%). A significant correlation was found between the preoperative risk score and these outcomes (Tables 3 and 4).

**Table 3:** Outcomes data for Group I and Group II according to the EuroSCORE II risk category

Variable			
	Low-risk (N = 3652)		
	Group I (ACHD) N = 356	Group II (non-ACHD) N = 3362	p-value
Operative mortality	2 (0.6)	56 (1.7)	0.110
ICU (days)	1 (1–2)	1 (1–3)	0.001
Mechanical ventilation (hours)	11 (7–16)	12 (8–17)	0.001
Hospital stays (days)	11 (8–14)	8 (7–10)	0.001
Major morbidity	34 (9.6)	362 (10.8)	0.479

Variable			
	Medium-risk (N = 912)		
	Group I (ACHD) N = 48	Group II (non-ACHD) N = 812	p-value
Operative mortality	1 (2.1)	37 (4.6)	0.418
ICU (days)	2 (1–5)	3 (1–5)	0.117
Mechanical ventilation (hours)	16 (12–18)	17 (12–22)	0.274
Hospital stays (days)	15 (11–21)	10 (7–14)	0.001
Major morbidity	13 (27)	192 (23.6)	0.587

Variable			
	High-risk (N = 489)		
	Group I (ACHD) N = 15	Group II (non-ACHD) N = 460	p-value
Operative mortality	1 (6.7)	77 (16.7)	0.300
ICU (days)	4 (2–7)	4 (2–9)	0.131
Mechanical ventilation (hours)	22 (16–60)	20 (16–63)	0.009
Hospital stays (days)	14 (11–27)	13 (8–21)	0.475
Major morbidity	5 (33.3)	176 (38.3)	0.699

Data are number (%) or mean (standard deviation) or median (interquartile range). ACHD: adult congenital heart disease. ICU: intensive care unit

**Table 4:** Outcomes data for Group I and Group II according to PEACH Score (ACHD) and EuroSCORE II (non-ACHD) risk category

Variable			
	Low-risk (N = 3652)		
	Group I (ACHD) N = 290	Group II (non-ACHD) N = 3362	p-value
Operative mortality	1 (0.3)	56 (1.7)	0.085
ICU (days)	1 (1–2)	1(1–3)	0.001
Mechanical ventilation (hours)	10 (6–14)	12 (8–17)	0.001
Hospital stays (days)	10 (8–14)	8 (7–10)	0.001
Major morbidity	21 (7.2)	362 (10.8)	0.071

Variable			
	Medium-risk (N = 912)		
	Group I (ACHD) N = 100	Group II (non-ACHD) N = 812	p-value
Operative mortality	1 (1)	37 (4.6)	0.112
ICU (days)	2 (1–3)	3 (1–5)	0.001
Mechanical ventilation (hours)	14 (9–18)	17 (12–22)	0.002
Hospital stays (days)	13 (9–18)	10 (7–14)	0.001
Major morbidity	21 (21)	192 (23.6)	0.618

Variable			
	High-risk (N = 489)		
	Group I (ACHD) N = 29	Group II (non-ACHD) N = 460	p-value
Operative mortality	2 (6.9)	77 (16.7)	0.201
ICU (days)	3 (1–7)	4 (2–9)	0.131
Mechanical ventilation (hours)	17 (13–20)	20 (16–63)	0.009
Hospital stays (days)	17 (11–26)	13 (8–21)	0.475
Major morbidity	10 (34.5)	176 (38.3)	0.844

Data are number (%) or mean (standard deviation) or median (interquartile range). ACHD: adult congenital heart disease. ICU: Intensive care unit

### Group II: Adult Patients with Acquired Heart Disease

A total of 4634 consecutive adult patients with acquired heart disease were operated on in our institution during the study period. Mean age was 67.7 years (+ −32 years).

Details on the type of surgery performed are provided in Table 1.

Coronary Artery By-pass Grafting (CABG) was the most frequent surgical procedure performed (44%), including 1298 isolated CABG, 556 CABG associated to valve surgery, and 185 CABG and LV aneurism resection. Single valve procedure was performed in 33% of cases, and multiple valve procedures in 8%. Surgery on the ascending aorta accounted for 5% of cases (Table 1).

According to the type of operation performed, 4312 patients (93%) had primary repair, while 322 patients (7%) had a reoperation after previous procedures (Fig. 1).

The preoperative risk stratification of patients in this Group was calculated using EUROSCORE II [15]. The risk stratification was performed as follows: < 2 = low risk; 2–6.99: medium risk; ≥ 7: high risk. 3362 patients were included in low-risk class (72%), 812 (17.5%) in medium risk class, and 460 patients (9.9%) in high-risk category (Fig. 2). Cumulative in-hospital mortality was 3.7% (n = 170 patients).

Median total postoperative hospital stay was 8 days (range 7–12 days) including: median ICU stay of 2 days (range 1–4 days), and median time of MV of 14 h (range 10–18 h) (Table 2). Major morbidity occurred in 730 patients (15%).

Comparative data on outcomes between Group I and Group II are shown in Table 2.

Univariate analysis revealed that patients with ACHD are younger (37.8 vs. 67.7 years), more frequently redo operations (32.1% vs. 6.9%), and have longer post-ICU hospital stay (11 vs. 8 days). However, ICU stay is lower (1 vs 2 days), with shorter assisted mechanical ventilation time (12 vs 14 h), and lower surgical mortality (1 vs 3.7%) (all p < 0.001).

The percentage of high-risk patients were 4% in Group I and 11% in Group II respectively.

No differences were found in term of post-operative complications (12.4 vs. 15%, p = 0.078).

Outcomes data for Group I and Group II according to the risk categories are shown in Tables 3 and 4.

Data are number (%). ACHD: adult congenital heart disease; CABG: coronary artery bypass grafting; LV: left ventricle; PA: pulmonary artery; RV: right ventricle.

## Discussion

Early diagnosis, advances in surgical and interventional technique, and medical therapy have increased overall survival to the adulthood of new-borns affected by moderate to complex CHD. The ACHD population is constantly growing and aging with a progressive increase of hospital admissions particularly in those with complex CHD [7, 19, 20]. In United States in the period 1998–2005 the number of patients hospitalized with complex CHD increased by 52.8% and the total charges for ACHD hospitalizations increase from 691 million to 3.16 billions of dollars [20]. Many of these patients need to be operated or re-operated, and there seems to be a general consensus that those with moderate to highly complex CHD, can be difficult to manage, have very special needs and consequently elevated costs. Adult patients with CHD are presumed to be high users of resources for health care [8, 9] and this, in a period of global economic crisis, can generate concern about the accessibility of care for them.

Few studies are present in the literature. Nasr et al. [8] showed that ACHD patients undergoing cardiac surgery experience higher hospital costs and poorer outcomes than a reference population of adult CABG patients related to higher complication rate and mortality, and a higher comorbidity index. Karangelis et al. [10] arrived to similar conclusions comparing 135 ACHD patients undergoing cardiac surgery over 3 consecutive years to 42 patients with structurally normal heart who had cardiac surgery for acquired heart disease. Collins II et al. [9] demonstrated that the costs of hospitalizations for ACHD patients with single ventricle and arrhythmias is 600% of that of adults without CHD admitted with a cardiac arrhythmia. Seckeler [11] showed that adults with moderate to severe CHD admitted to the hospital for non-cardiac diagnoses had longer hospitalization and ICU stay compared to adults without CHD. Recently the paper from Williamson et al. [12] demonstrated that the presence of CHD is a risk factor that portends unfavourable outcomes in ACHD undergoing major elective non-cardiac operations. Finally, Setton et al. [13] analysing 16,841 ACHD surgery admission demonstrated that complications occurred frequently in these patients with increased resource use.

But are these few studies a real and current picture of the complexity of ACHD patients and the difficulties of their management?

Heart surgery for congenital and acquired heart disease has dramatically changed over time, and a better understanding of the actual complexity of these patients is mandatory, in current era, for the management of resources and to decide the most appropriate hospital setting [21].

While an extensive amount of evidence on surgical outcomes for acquired heart disease is available and demonstrates the profound change in complexity occurred in the last few years [22–24], the majority of studies involving ACHD patients accounts for US data regarding general costs and insurance access [8–11, 19, 20, 25–29]. On the other hand, reports focused on outcomes of congenital cardiac surgery mainly concern paediatric patients [30–32], not including the ACHD population. Conclusions from the previously reported researches agree that the higher costs in cardiac surgery are related to the occurrence of postoperative complications, and the length of in-hospital and ICU stay [8–11, 20].

ACHD patients are a very heterogeneous population with such a diverse spectrum of anatomy, physiology, and different adjustments to potential residual hemodynamic sequelae and our study is one of the first in Europe, to our knowledge, to compare the outcomes of cardiac surgery in ACHD with the population of adult with acquired heart disease. Our experience provides a current and broad overview, from a large volume tertiary centre, that could be potentially useful for a wider cardiac/cardiological community.

Our comparative analysis of the two groups (Group I-ACHD, Group II-non ACHD) reveals interesting information, somewhat in contrast with the previously published reports.

Congenital and acquired adult patients with cardiac disease present some substantial differences, some of which are easily understandable such as age and the type of pathology.

In Group I the pathology of the RVOT was the most frequently treated while CABG (isolated or associated with other surgical procedures) was the most frequent surgery performed in Group II.

Patients of Group I are significantly younger and more frequently had one or more previous operations. Group I have lower surgical mortality, they stay longer in hospitals, but have a shorter ICU stay and shorter assisted mechanical ventilation time.

The longest post-ICU stay can be explained by the fact that, in our institution, acquired patients are usually quickly transferred to rehabilitation centers on a regular basis, while ACHD patients are most often discharged home and therefore remain longer in the hospital. Furthermore, we have different departments for ACHD and non ACHD patients with full-time dedicated cardiologists. Therefore, there are two separate management approaches for the analyzed groups, which could potentially partially justify the length of the hospital stay.

The lower ICU stay, and mechanical ventilation time observed in Group I can be explained by the fact that, from the analysis of the surgical procedures and distribution of risk categories (Table 1 and Fig. 2), 93% of the ACHD patients were in the low or medium risk class, foreseeing short recovery times in substantially young subjects, with fewer comorbidities. Complex procedures such as Fontan conversion/revision, congenital redo AV valve procedures or Bentall operation, in fact, represent only 7% of the total.

The reduced number of high-risk patients in Group I, associated with the increasing experience of their management, may explain the excellent results that can be obtained in a high-volume tertiary center.

We believe that this study can provides important information for health care professionals, administrators, medical insurances, National Health Services, and patient’s associations.

ACHD patients requiring cardiac surgery must be considered a heterogeneous group of patients where only a limited proportion of patients are high risk. The majority of them (more than 90%), in fact, fits in the low or medium risk category, resulting in better outcomes (mortality, ICU stay, assisted mechanical ventilation time, and post-operative complications), compared to a reference population of patients with acquired cardiac disease.

## Limitations

There are several limitations in this study. Firstly, this study is retrospective in nature and subject to limitations inherent to observational investigations and retrospective data collection. Secondly, the study is restricted to our experience from a single centre. Finally, we compared two groups of different patients from both the numerical and clinical point of view.

## Conclusions

The population of adults affected by congenital heart disease is constantly growing. The previous limited data available suggested that these patients could be difficult to manage, have very special needs, elevated costs, and worse outcomes. Our study shows that ACHD patients requiring cardiac surgery must be considered a heterogeneous group, where only a limited number of patients falls in the high-risk category. In a high-volume tertiary centre, adult patients with CHD undergoing cardiac surgery have excellent results, experience lower ICU stay, lower assisted mechanical ventilation time, and lower mortality compared to a reference population of adult patients with acquired cardiac disease.
